# PINK1 expression increases during brain development and stem cell differentiation, and affects the development of GFAP-positive astrocytes

**DOI:** 10.1186/s13041-016-0186-6

**Published:** 2016-01-08

**Authors:** Insup Choi, Dong-Joo Choi, Haijie Yang, Joo Hong Woo, Mi-Yoon Chang, Joo Yeon Kim, Woong Sun, Sang-Myun Park, Ilo Jou, Sang Hoon Lee, Eun-Hye Joe

**Affiliations:** Neuroscience Graduate Program Department of Biomedical Sciences, Ajou University School of Medicine, Suwon, Korea; Chronic Inflammatory Disease Research Center, Ajou University School of Medicine, Suwon, Korea; Department of Pharmacology, Ajou University School of Medicine san-5, Woncheon-dong, Youngtong-gu, Suwon, Kyunggi-do, 442-721 Korea; Department of Biochemistry and Molecular Biology, College of Medicine, Hanyang University, Seoul, Korea; Department of Anatomy and Division of Brain Korea 21 Plus Biomedical Science, Korea University College of Medicine, Seoul, 136-705 Korea; Department of Brain Science, Ajou University School of Medicine, Suwon, Korea; Brain Disease Research Center, Ajou University School of Medicine, Suwon, Korea

**Keywords:** PINK1, Neural stem cell, Astrocyte, Parkinson’s disease

## Abstract

**Background:**

Mutation of PTEN-induced putative kinase 1 (PINK1) causes autosomal recessive early-onset Parkinson’s disease (PD). Despite of its ubiquitous expression in brain, its roles in non-neuronal cells such as neural stem cells (NSCs) and astrocytes were poorly unknown.

**Results:**

We show that PINK1 expression increases from embryonic day 12 to postnatal day 1 in mice, which represents the main period of brain development. PINK1 expression also increases during neural stem cell (NSC) differentiation. Interestingly, expression of GFAP (a marker of astrocytes) was lower in PINK1 knockout (KO) mouse brain lysates compared to wild-type (WT) lysates at postnatal days 1-8, whereas there was little difference in the expression of markers for other brain cell types (e.g., neurons and oligodendrocytes). Further experiments showed that PINK1-KO NSCs were defective in their differentiation to astrocytes, producing fewer GFAP-positive cells compared to WT NSCs. However, the KO and WT NSCs did not differ in their self-renewal capabilities or ability to differentiate to neurons and oligodendrocytes. Interestingly, during differentiation of KO NSCs there were no defects in mitochondrial function, and there were not changes in signaling molecules such as SMAD1/5/8, STAT3, and HES1 involved in differentiation of NSCs into astrocytes. In brain sections, GFAP-positive astrocytes were more sparsely distributed in the corpus callosum and substantia nigra of KO animals compared with WT.

**Conclusion:**

Our study suggests that PINK1 deficiency causes defects in GFAP-positive astrogliogenesis during brain development and NSC differentiation, which may be a factor to increase risk for PD.

**Electronic supplementary material:**

The online version of this article (doi:10.1186/s13041-016-0186-6) contains supplementary material, which is available to authorized users.

## Background

PTEN-induced putative kinase 1 (PINK1) is a PD-related gene whose mutation causes an autosomal recessive early-onset PD [1]. PINK1 plays diverse roles. For example, it regulates mitochondrial function [2], which is linked to ATP generation, oxygen consumption [3–5], and ROS production [6]. In addition, PINK1 regulates the AKT-mTOR and HIF-1 alpha pathways to mediate proliferation, survival, metabolism, and inflammation etc. [7–12]. Finally, PINK1 deficiency reportedly reduces astrocyte proliferation [7] and neurite outgrowth [13], suggesting that this deficiency may affect brain development and/or injury repair.

Astrocytes, which are the most abundant cells in the brain, express glial fibrillary acidic protein (GFAP) and are known to play important roles in developing, intact, and injured brains. Astrocytes regulate synaptogenesis [14], neural activity, and neural circuit formation in both developing and injured brains [15–17]. In intact brain, astrocytes support neurons by providing nutrients and growth factors [18–20], and maintaining the homeostasis of extracellular potassium and glutamate [21, 22]. In injured brain, astrocytes become hypertrophic, exhibit increased GFAP expression, and proliferate, thereby isolating injury sites, preventing oxidative stress and neuronal death, and decreasing inflammation [23–28]. The neural stem cells (NSCs) in the subventricular zone (SVZ) of the brain are a specialized form of GFAP-expressing astrocytes [29] that contributes to injury repair. In ischemic brain, it was recently reported that astrocytes differentiate into new neurons and participate in regenerating the injured brain [30]. Therefore, defects of astrogliogenesis could cause brain abnormalities, including neurodegeneration [31].

In this study, we show that PINK1 expression increases during brain development and NSC differentiation, whereas PINK1 deficiency decreases GFAP expression during these processes. Subsequent experiments revealed that PINK1 deficiency causes defects in astrogliogenesis, decreasing the number of GFAP-positive astrocytes and causing abnormalities in their locations and configurations in the corpus callosum, and substantia nigra reticulate. Collectively, these findings suggest that defects in GFAP-positive astrogliogenesis could be a mechanism through which PINK1 deficiency could contributes to the development of PD.

## Results

### The expression levels of PINK1 increase during brain development, and GFAP expression is attenuated in PINK1-deficient mouse brains

**Fig. 1:**
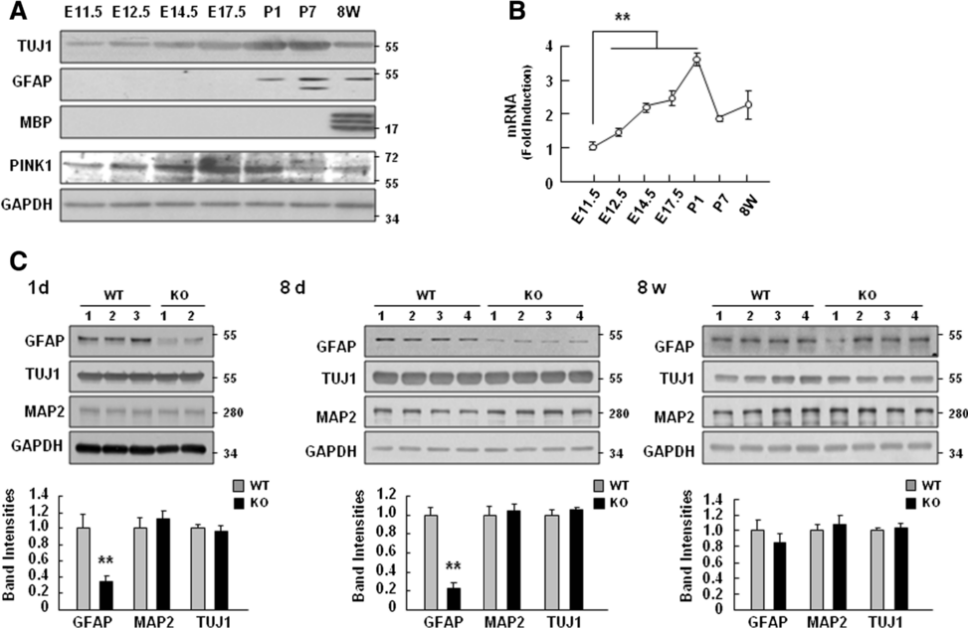
PINK1 expression increased during brain development, and PINK1 deficiency caused defects in GFAP expression. **a**, **b** Mouse brains were collected at the indicated ages. The levels of a neuron marker (TUJ1), an astrocyte marker (GFAP), an oligodendrocyte marker (MBP), and PINK1 were assayed by Western blotting, with GAPDH used as the loading control (**a**). The mRNA levels of PINK1 during brain development were determined using Q-PCR (**b**). **c** At postnatal day 1, postnatal day 8 and 8 weeks, whole brains from WT and PINK1-KO mice were collected and GFAP, TUJ-1, and MAP2 protein levels were analyzed by Western blotting. The band intensities of GFAP, TUJ1 and MAP2 were quantified and normalized with respect to that of GAPDH. The data shown are representative of two independent experiments (**a**, **b**). Values in (**b**, **c**) are means ± SEMs of at least four samples (**, *P* < 0.01)

Since PINK1 is closely associated with the signaling pathways that regulate cell proliferation, survival, and differentiation [7–9], we first examined the expression levels of PINK1 during brain development during a period characterized by the vigorous proliferation and differentiation of brain cells. Brain lysates were prepared from samples taken on embryonic day 11.5 (E11.5) through E17.5, as well as on postnatal day 1 (P1), P7, and at 8 weeks after birth. The protein expression of the neuronal marker, TUJ-1, gradually increased from E11.5 to adulthood and, as previously reported [32], the astrocyte marker, GFAP, appeared at around P1 (Fig. 1a). The oligodendrocyte marker, myelin basic protein (MBP), was not detected up to P7, but could be detected at 8 weeks (Fig. 1a). Interestingly, the PINK1 protein expression levels showed some correlation with brain development, increasing from E11.5 to a peak at E17.5 and P1, and then decreasing at P7 and 8 weeks (Fig. 1a). The mRNA levels of PINK1 showed a similar expression pattern, gradually increasing from E11.5 to a peak at P1, and then decreasing at P7 and 8 weeks (Fig. 1b). We also found that protein expression of Parkin, another PD gene [33], showed similar patterns to that of PINK1 (Additional file 1: Figure S1).

Since PINK1 expression was upregulated during brain development, particularly during the period when the expression levels of TUJ1 and GFAP were also increased (Fig. 1a, b), we questioned whether PINK1 could be functionally associated with the expression levels of TUJ1 and/or GFAP. Accordingly, we compared the expression levels of GFAP, TUJ1, and MAP2 (another marker of neurons) in WT and PINK1-knockout (KO) brains at P1, P8, and 8 weeks. Using Western blot, we confirmed absence of PINK1 protein expression in PINK1 KO mice (Additional file 2: Figure S2). The PINK1 deficiency in PINK1-KO mice was usually confirmed using genotyping prior to the preparation of brain lysates as previously described [34]. Interestingly, we found that GFAP protein levels in PINK1-KO brain were lower than in WT brain at P1 and P8, whereas there was little difference in the levels of TUJ1 and MAP2 (Fig. 1c). At 8 weeks, however, there was no significant difference in the levels of GFAP as well as TUJ1 or MAP2 in WT and PINK1-KO brains (Fig. 1c). These results suggest that PINK1 regulates brain development, particularly, GFAP expression.

### The expression levels of PINK1 increase during NSC differentiation, and GFAP expression is attenuated in PINK1-deficient NSCs in vitro

**Fig. 2:**
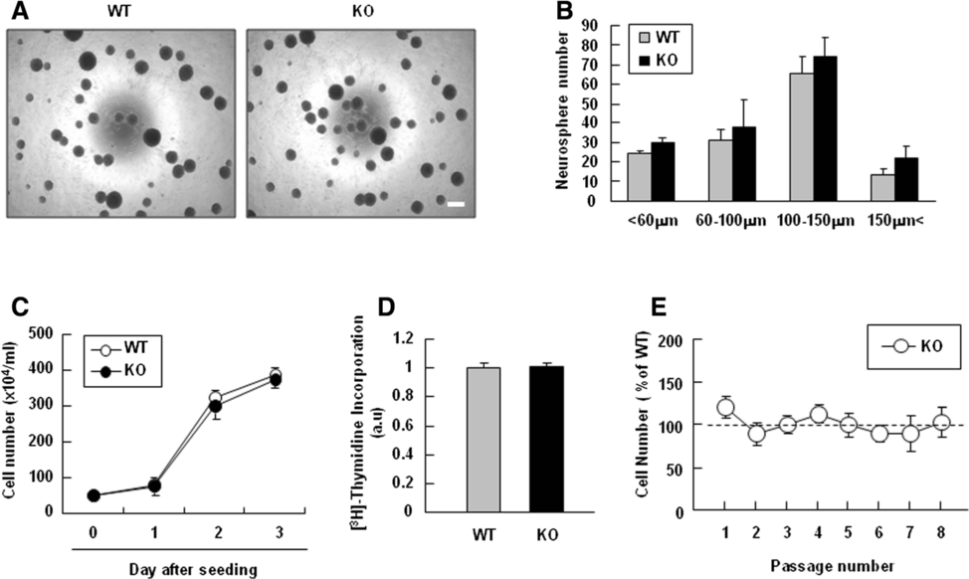
PINK1 deficiency did not affect the self-renewal or proliferation capacities of NSCs. **a**, **b** The secondary neurosphere formation ability was assayed in WT and PINK1-KO NSCs. Dissociated primary neurospheres (2 × 10^4^ cell/well) were seeded to a 96-well plate in the presence of EGF (20 ng/ml) and FGF (20 ng/ml). On day 3 of seeding, the sizes and numbers of secondary neurospheres were analyzed. **c**, **d** NSC proliferation was determined by counting of cell numbers (**c**) and measurement of [^3^H]-thymidine incorporation (**d**). Dissociated NSCs were plated to 24-well plates coated with poly-L-ornithine and fibronectin (1 × 10^5^ cell/well) in the presence of EGF and FGF. On the following day, 1 μCi/ml [^3^H]-thymidine was added with growth factors, and the plates were incubated for an additional 24 h. Finally, the NSCs were washed with PBS and lysed with 0.1 N NaOH, and radioactivity was determined using a β-counter. **e** WT and PINK1-KO NSCs were subcultured every 3-4 days after neurosphere formation in the presence of 20 ng/ml EGF and FGF until passage 8. At each passage, the number of cells was counted. Values in (**b**, **c**) are means ± SEMs of at least three samples. Scale bar in (**a**), 200 μm. The data shown are representative of at least three independent experiments

Since GFAP expression differed in WT and KO during brain development (Fig. 1c), we examined whether PINK1 regulates the proliferation and/or differentiation of NSCs obtained from E13.5 mouse brains. The NSCs were cultured as neurospheres, and their proliferative capacity was assessed by counting the number and size of secondary neurospheres, measuring [^3^H]-thymidine incorporation, and assessing the cell numbers. We previously reported that PINK1 regulates astrocyte proliferation [7]. However, proliferation defect was not found in PINK1-KO NSCs since the number and size of neurospheres derived from WT and PINK1-KO NSCs were similar (Fig. 2a and b). Additionally, the cell numbers and [^3^H]-thymidine incorporation level did not significantly differ between WT and PINK1-KO NSCs (Fig. 2c and d), and the proliferation capacities of WT and PINK1-KO NSCs did not significantly differ even at a later passage (passage 8) (Fig. 2e).

**Fig. 3:**
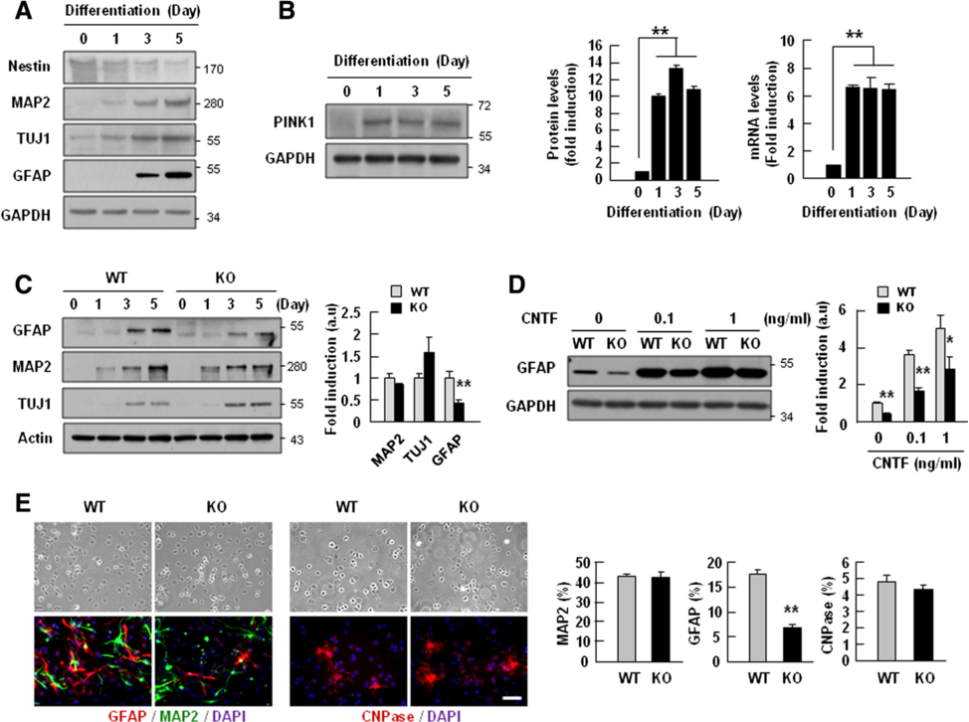
PINK1 expression increased during differentiation of NSCs, and its deficiency caused defects in GFAP-positive astrocyte differentiation. NSCs (1.5 × 10^6^ cell/well) were prepared from E13.5 WT and PINK1-KO mouse embryo brain and seeded to 6-well plates coated with poly-L-ornithine/fibronectin. Differentiation was induced by withdrawal of EGF and FGF. **a**, **b** At the indicated times after induction of differentiation, protein levels of Nestin, MAP2, TUJ-1, and GFAP (**a**), and protein and mRNA levels of PINK1 (**b**) were assayed, and the results were quantified (**b**). **c** The differentiation capacities of WT and PINK1-KO NSCs into astrocytes (GFAP) and neurons (TUJ-1 and/or MAP2) were analyzed by Western blot (left panel) and quantified (right panel). **d** The differentiation capacities of WT and PINK1 KO NSCs into astrocytes (GFAP) were analyzed in the presence of CNTF by Western blot (left panel) and quantified (right panel) on day 5 of differentiation. GAPDH or actin was used as the loading control. **e** On day 5 of differentiation, cells were immunostained for cell-type-specific markers, neuron (MAP2), astrocyte (GFAP), and oligodendrocytes (CNPase) (left panel). Images were taken using a Zeiss microscope, and the number of each type of cells was counted using the Image J software, and plotted (right panel). Scale bar in (**e**), 50 μm. Values in (**b**, **c**, **d**, and **e**) are means ± SEMs of at least three samples (**, *P* < 0.01). The data shown are representative of at least three independent experiments

We further examined whether PINK1 regulates NSC differentiation. Interestingly, the mRNA and protein levels of PINK1 significantly increased during differentiation into neurons and astrocytes, as demonstrated by increases in MAP2, TUJ1, and GFAP after 1–5 days of differentiation, and a decrease in nestin (an NSC marker) expression beginning after 1 d of differentiation (Fig. 3a, b). As in developing brain, Parkin protein expression also increased during differentiation of NSCs similar to PINK1 protein expression (Additional file 3: Figure S3).

We next compared the differentiation patterns of WT and PINK1-KO NSCs. During the induction of NSC differentiation, the protein levels of MAP2 and TUJ1 were similar in WT and PINK1-KO (Fig. 3c). However, GFAP protein levels were significantly lower in PINK-1 KO cells compared to WT NSCs on days 3 and 5 of differentiation (Fig. 3c). The decrease was not due to cell death, as indicated by similar levels of cleaved PARP, cleaved caspase-3, and LDH between WT and PINK1-KO NSCs (Additional file 4: Figure S4). We also examined differentiation of NSCs in the presence of CNTF, a well known strong inducer of astrocyte differentiation [35]. On day 5 of differentiation, CNTF dose-dependently (in the range of 0.1–1 ng/ml) increased the differentiation of NSCs into astrocytes, as demonstrated by GFAP expression (Fig. 3d). Furthermore, the GFAP protein level was lower in PINK1-KO NSCs than in WT NSCs (Fig. 3d). Immunostaining with antibodies specific for GFAP, MAP2 and CNPase revealed that there were significantly fewer GFAP-positive cells among PINK1-KO NSCs compared to WT NSCs on day 5 of differentiation (17.7 % vs. 6.8 %), whereas the numbers of MAP2- (43.1 % vs. 42.4 %) and CNPase-positive cells (4.8 % vs. 4.3 %) were not significantly different (Fig. 3e). Collectively, these results suggest that PINK1 is required for the differentiation of NSCs into GFAP-positive astrocytes.

### Neither GFAP mRNA expression nor signaling pathways involved in gliogenesis are changed in PINK1-deficient NSCs

**Fig. 4:**
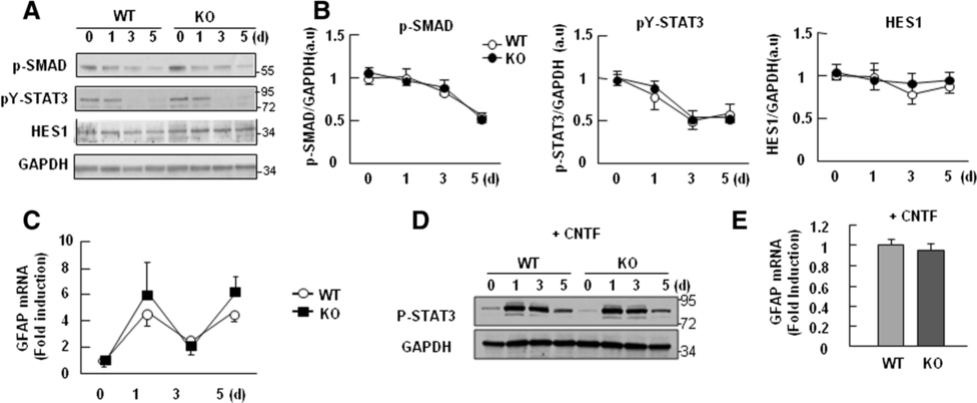
PINK1 deficiency did not affect GFAP mRNA expression or the signaling pathways involved in astrocyte differentiation. **a**, **b** The levels of proteins known to be involved in astrocyte differentiation-related pathways (e.g., p-SMAD1/5/8, p-STAT3, and HES1) were measured by Western blotting (**a**). GAPDH was used as the loading control. The band intensities of p-SMAD1/5/8, p-STAT3, and HES1 were quantified in (**b**). **c** GFAP mRNA levels were measured in WT and PINK1-KO NSCs using Q-PCR on days 0, 1, 3, and 5 of differentiation. **d**, **e** WT and PINK1-KO NSCs were differentiated in the presence of 0.1 ng/ml CNTF for 5 days. p-STAT3 levels were compared after the indicated durations of differentiation (**d**), and GFAP mRNA levels were examined on day 3 of differentiation (**e**). Values are means ± SEM of at least four samples (**b**, **c**, **e**)

In an effort to identify the mechanisms responsible for decreasing the differentiation of PINK1-KO NSCs into GFAP-positive astrocytes, we examined the activation levels of the signaling molecules involved in astrogliogenesis, including STAT3 [36–38], SMAD1/5/8 [39], and HES1 [40]. The activation of these molecules are evaluated by phosphorylation (SMAD1/5/8 and STAT3) [41, 42], or expression (HES1) [43]. Unexpectedly, however, there was no difference in the levels of pSMAD1/5/8, pSTAT3, and HES1 (Fig. 4a, b), even in the presence of CNTF (Fig. 4d). Accordingly, mRNA levels of GFAP did not differ significantly in WT and PINK1-KO NSCs during differentiation in the absence (Fig. 4c) and presence of CNTF (Fig. 4e), suggesting that PINK1 dose not regulate GFAP expression at transcriptional level.

In further studies, we examined the effect of proteasome inhibitors, MG132 and lactacystin, on GFAP expression. However, these inhibitors also had little effect on GFAP expression (Additional file 5: Figure S5), suggesting that PINK1 did not alter the protein stability of GFAP. Therefore, further studies are required to assess how PINK1 regulates GFAP expression and/or the generation of GFAP-positive astrocytes.

### Mitochondrial defects were not found in PINK1 deficient NSCs during differentiation

**Fig. 5:**
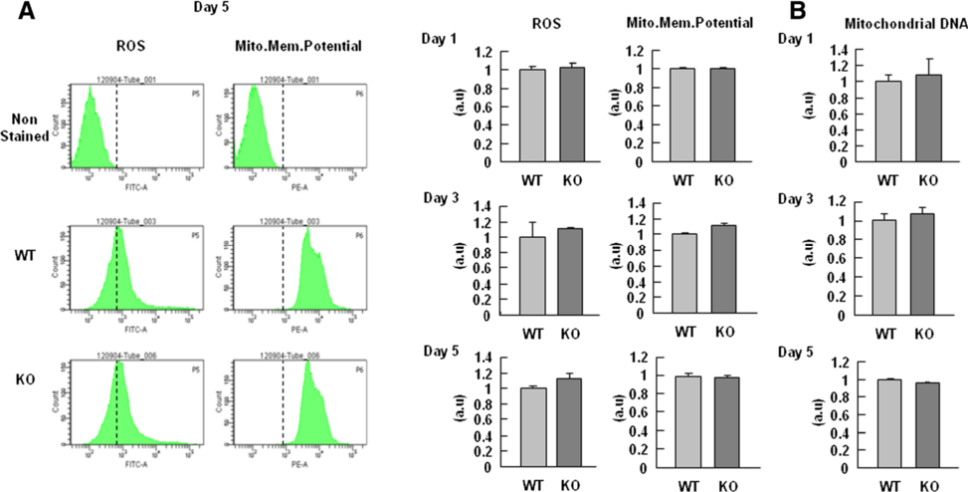
PINK1-KO NSCs exhibited normal mitochondrial functions during differentiation. **a** On day 5 (*left panel*) or the indicate times of differentiation (*right panel*), intracellular ROS levels and the mitochondrial membrane potential were monitored by loading cells for 30 min with 10 μM carboxyl-H2DFFDA and 125 nM MitoTracker Red CMXRos, respectively. **b** On days 1, 3, and 5 of differentiation, the content of mitochondrial DNA relative to that of nuclear DNA was measured as the ratio of the mitochondrial D-loop (mito-D-loop) to the nuclear-encoded GAPDH gene, using Q-PCR as described in Methods. Data are means ± SEM of three samples. The data shown are representative of at least three independent experiments

Next, we examined the possible involvement of mitochondrial dysfunction in abnormal astrogliogenesis in PINK1 deficient NSCs, since we and others have found that PINK1 deficiency causes mitochondrial dysfunctions in neurons and astrocytes [2, 7, 44]. However, mitochondrial dysfunction was not detectable in PINK1 KO NSCs for up to 5 days after the induction of differentiation. WT and KO NSCs did not significantly differ in their mitochondrial membrane potential or ROS production, as measured by FACS analysis using MitoTracker Red CMXRos and carboxyl-H_2_DFFDA, respectively (Fig. 5a). In addition, the mitochondrial DNA copy number did not differ between WT and PINK1-KO NSCs (Fig. 5b). These findings suggest that PINK1 may not be required for normal mitochondrial function in NSCs differentiation.

### Differences in the distribution of GFAP-positive cells in the lateral ventricle and/or substantia nigra (SN) of WT and PINK1-KO mice

**Fig. 6:**
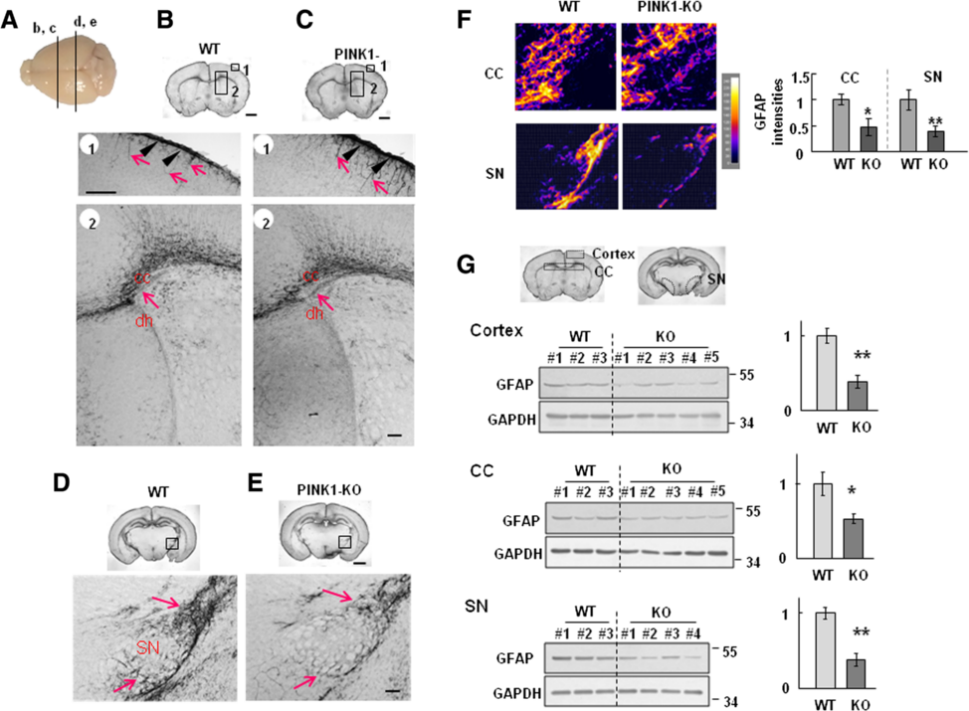
PINK1 deficiency alters GFAP expression in the cortex and midbrain of 8-day-old mice. Coronal sections were obtained from 8-day-old WT and PINK1 KO mice, stained with GFAP antibodies, and visualized with peroxidase-conjugated secondary antibodies. **a** Positions of sections presented in (b-e) are indicated. **b**, **c** In the cortex, the pia mater was strongly stained with anti-GFAP antibodies in both WT and KO sections (arrowheads in b1 and c1, respectively). The GFAP-positive processes underneath the pia mater were thinner in WT samples (arrows in b1) than in KO samples (arrows in c1). The region where the dorsal horn (dh) of the lateral ventricle connected to the corpus callosum (CC) was strongly immunoreactive for GFAP in WT sections but not in KO sections (arrows in b2 and c2, respectively). **d**, **e** The SN in the midbrain of WT mice was less compactly filled with GFAP positive processes in KO sections compared with WT sections (arrows in d and e, respectively). Images were captured by a microscope (Zeiss). **f** Images were analyzed using Image J. Scale bar, 1 mm (upper panel in b-e), 100 μm (middle and lower panel in b-e). **g** Brain lysates were prepared from each region (cortex, CC: corpus callosum, SN: substantia nigra) shown in the above panel, and GFAP levels were analyzed with western blot. Each number indicates different animal (*left panel*). Band intensities were measured and plotted (*right panel*). The data shown are representative of at least three different animals. Values in (**f** and **g**) are means ± SEM of four samples. (*, *P* < 0.01; **, *P* < 0.01)

Next, we analyzed GFAP-positive astrocytes in several regions of WT and PINK1-KO mouse brains, including the lateral ventricles (Fig. 6a, b, c) and SN, where dopaminergic neuronal processes and cell bodies locate (Fig. 6a, d, e). In the cortex of P8 mice, GFAP immunoreactivity was detectable in the pia mater (arrowheads in Fig. 6b1 and c1) and the thin processes beneath this structure (arrows in Fig. 6b1 and c1), but these processes were thicker and longer in PINK1-KO brains (arrows in Fig. 6b1 and c1). Interestingly, the morphology and/or distribution of GFAP-positive astrocytes in WT and KO mice differed in the corpus callosum (CC); in particular, the point at which the dorsal horn (dh) of the lateral ventricle (which was not yet fully developed at this stage) connected to the CC was filled with GFAP-immunoreactive cells in WT but not in KO mice (arrows in Fig. 6b2 and c2). Finally, the P8 SN was densely populated with GFAP-immunoreactive astrocytes in WT brains, but only sparsely populated with these astrocytes in KO brains (arrows in Fig. 6d and e). Image analysis using Image J (f) and western blot using brain lysates prepared from each brain regions (g) showed decrease in GFAP expression in PINK1 KO brain. Taken together, these results indicate that GFAP-positive astrocytes developed abnormally in PINK1-deficient mouse brains and NSCs.

## Discussion

The results of this study show that PINK1 expression increases during brain development and NSC differentiation, and that this increase is related to changes in GFAP expression during these two processes. Furthermore, PINK1 deficiency decreased the differentiation of NSCs into GFAP-positive astrocytes, and caused defects in the location and/or distribution of GFAP-positive astrocytes in the SVZ and/or SN.

In injured brain, SVZ-NSCs migrate toward injury sites and differentiate into astrocytes as well as neurons [45–49]. Astrocytes contribute to restoring disrupted extracellular fluid homeostasis and repairing the injured brain: astrocytes increase expression of glutamate and potassium transporters [25, 26], facilitate axon regeneration [50–52], constitute a part of the neurogenic niche [53–55], and affect neurogenesis [56, 57]. Accordingly, in ischemic brain, disruption of the differentiation of SVC-NSCs to astrocytes induces abnormal astrogliosis, which results in an exaggerated microvascular hemorrhage [46]. Therefore, defects in astrogliogenesis and/or astrocyte functions can decrease neuronal support and impair the repair of injured brain, potentially leading to gradual neuronal death and accumulation of damage, which results in neurodegenerative diseases [31, 58–61].

Next arising question was how PINK1 decreases differentiation of PINK1-KO NSCs into GFAP-positive astrocytes. We excluded the possible involvement of cell death in the decreased differentiation of PINK1-KO NSCs into GFAP-positive astrocytes, as assessed by the amounts of cleaved PARP, cleaved caspase-3, and LDH release (Additional file 4: Figure S4). Additionally, PINK1 deficiency did not switch the balance of NSC differentiation from neurogenesis to gliogenesis, since the number of TUJ-1-positive cells did not increase (Fig. 3c, e). During differentiation of WT and PINK1-KO NSCs, mRNA levels of GFAP did not differ significantly (Fig. 4c), and the activation levels of the signaling pathways involved in gliogenesis, such as STAT3 [36–38], SMAD1/5/8 [39], and HES1 [40] were also little different (Fig. 4b). Furthermore, mitochondrial dysfunction that has been found in PINK1 deficient neurons and astrocytes [2, 7, 44] was not detectable in PINK1-KO NSCs before and after the induction of differentiation (Fig. 5). Although mitochondrial dysfunction retarded proliferation of PINK1 deficient astrocytes [7], the proliferation of PINK1-KO NSCs may be normal based on their normal mitochondrial function (Fig. 2). These findings suggest that PINK1 may not be required for normal mitochondrial function in NSCs differentiation and/or that other genes may substitute for PINK1 in this case. It is also possible that PINK1-induced mitochondrial defects may accumulate in an age-dependent manner. Since GFAP mRNA expression was not reduced at PINK1-KO NSCs, we further examined the effect of proteasome inhibitors, MG132 and lactacystin, on GFAP expression (Additional file 5: Figure S5). Interestingly, these inhibitors had little effect, suggesting that PINK1 did not alter the protein stability of GFAP. Recently, it has been reported that several PD genes may regulate protein translation [62]. Therefore, further studies should be done to assess whether PINK1 may regulate GFAP expression at translation levels.

The importance of glia in the maintenance of brain function is beyond question, and their loss and/or abnormal function can contribute to neurodegeneration [63]. Our group and others have reported that mutations in several PD genes can alter the functions of astrocytes and other brain cells, including NSCs and microglia. For example, mutation of DJ-1 attenuates the neuroprotective functions of astrocytes [64]. Studies have shown that the inflammation and endocytosis of astrocytes and microglia can be regulated by DJ-1, PINK1, and LRRK2 [65–67], while the proliferation capacity of astrocytes is regulated by PINK1 [7], and LRRK2 mutation affects the viability of stem cells [68]. In this study, we found that Parkin similar to PINK1 changed its expression during development of the brain and NSC differentiation (Additional file 1: Figure S1, Additional file 3: Figure S3) although both Parkin and PINK1 in monkey are decreased or remain unchanged during aging [69]. Taken together, these lines of evidence suggest that PD does not affect only neurons, but rather is also a disease of other brain cells, including astrocytes and NSCs.

## Conclusion

In conclusion, we herein provide the first evidence that PINK1 deficiency causes defects in the differentiation of NSCs to astrocytes and/or delay in GFAP expression and/or development of GFAP-expressing cells. Since astrocytes play critical roles in neuronal survival and the repair ininjured brain, insufficient astrocytic support due to PINK1 deficiency may cause neuronal death and/or abnormal tissue repair of the injured brain, accumulating damage and increasing the risk of PD. These possibilities imply that neurodegenerative diseases, including PD, could be diseases of astrocytes as well as neurons. Therefore, the functional regulation of non-neuronal cells should be a new target for the development of therapies for PD.

## Methods

### Animals

The PINK1-deficient mice were a generous gift from Dr. Xiaoxi Zhuang (Chicago University) and Dr. UJ Kang (Columbia University), and were as previously described [7]. All animal procedures were approved by the Ajou University School of Medicine Ethics Review Committee for Animal Research (Amc-119).

### Neurosphere culture and cell counting

Embryonic neurospheres were cultured from the brains of embryonic day 13.5 (E13.5) mice, as previously described [70]. Briefly, forebrains were freed of meninges and gently triturated several times in culture medium using a flame-polished Pasteur pipette. Cells from a single brain were plated in a 100-mm Petri dish and cultured in Dulbecco’s modified Eagle’s medium (DMEM)/F12 medium (WelGene, Daegu, Korea) supplemented with N-2, B27 supplement (Gibco-Invitrogen, Carlsbad, CA, USA), 20 ng/ml EGF, and bFGF (BD Bioscience, San Jose, CA, USA). EGF and bFGF were added every 2 days. For serial neurosphere formation, primary neurospheres were collected, incubated with Accumax (Millipore), and dissociated. For differentiation, dissociated cells were seeded on plates coated with 0.2 mg/ml poly-L-ornithine and 1 μg/ml fibronectin (Sigma) in the absence of growth factors or in the presence of CNTF (BD Bioscience).

For proliferation assays, dissociated primary neurospheres (2×10^4^ cell/well) were seeded to a 96-well plate and incubated in the presence of EGF and bFGF for 3 days, and the sizes and numbers of secondary neurospheres were analyzed using the TINA software (Raytest, Straubenhardt, Germany). For the cell counting and [^3^H]-thymidine incorporation assays, dissociated primary neurospheres (1 × 10^5^ cell/well) were seeded to a poly-L-ornithine- and fibronectin-coated 24-well plate in the presence of growth factors (added daily to prevent NSC differentiation). For cell counting, on the indicated day, adherent NSCs were incubated with Ca^2+^/Mg^2+^-free HBSS for 20 min, detached by pipetting, and counted. For the thymidine incorporation assay, 1 μCi/ml [^3^H]-thymidine was added on day 1 of culture. After 24 h, the adherent NSCs were washed three times with PBS and lysed with 0.1 N NaOH. Radioactivity was determined using a β-counter (Packard Instruments, Downers Grove, IL, USA).

### Western blot analysis

Cells and mouse brains were lysed on ice in RIPA buffer (50 mM Tris–HCl, pH 7.4, 1 % NP-40, 0.25 % Na-deoxycholate, 150 mM NaCl, 1 mM Na_3_VO_4_, and 1 mM NaF) containing protease inhibitors (2 mM phenylmethylsulfonyl fluoride [PMSF], 10 μg/ml leupeptin, 10 μg/ml pepstatin, and 2 mM EDTA) and a phosphatase inhibitor cocktail (GenDEPOT, Barker, TX, USA). Proteins were separated by SDS-PAGE, transferred to nitrocellulose membranes, and identified using specific antibodies. The antibodies for PINK1 (Cat. NO. 23707), MAP2, and MBP were obtained from Abcam (Cambridge, MA, USA); for Parkin, p-SMAD1/5/8, and p-STAT3 from Cell signaling technology (Danvers, MA, USA); for nestin and CNPase from Millipore (Bedford, MA, USA); for TUJ-1 from Covance (Berkeley, CA, USA); for GFAP from Sigma (Cat. No. G3893; St. Louis, MO, USA); and for HES1 and GAPDH from Santa Cruz Biotechnology (Santa Cruz, CA, USA). Membranes were incubated with peroxidase-conjugated secondary antibodies (Jackson Immuno Research, West Grove, PA, USA), and visualized with an enhanced chemiluminescence system (Daeil Lab Inc., Seoul, Korea).

### Q-PCR

Total RNA was isolated using RNAzol B (iNtRON, Sungnam, Korea), and cDNA was prepared using Avian Myeloblastosis Virus reverse transcriptase (Promega, Madison, WI, USA) according to the manufacturer’s instructions. The relevant mRNA levels were measured using a KAP SYBR FAST qPCR kit (Kapa Biosystems, Boston, MA, USA) and a RotoGene thermocycler (Corbett Research, Sydney, Australia). The primer pairs used in this study were synthesized by Integrated DNA Technologies (Coralville, IA, USA) and were as follows: PINK1, 5’-GCTTGCCAATCCCTTCTATG-3’ (sense) and 5’-CTCTCGCTGGAGCAGTGAC-3’ (antisense); GFAP, 5’-AGCTAGCCCTGGACATCGAGA-3’(sense) and 5’-GGTGAGCCTGTATTGGGACAA-3’(antisense); GAPDH (reference housekeeping gene), 5’-GCCTTCCGTGTTCCTACC-3’ (sense) and 5’-CCTCAGTGTAGCCCAAGATG-3’ (antisense). The cycle thresholds (Ct) for the PINK1 and GFAP gene transcripts were normalized to the average Ct for GAPDH, and the relative quantitation of normalized transcript abundance was determined using the comparative Ct method (ΔΔCt), as described by the manufacturer (Kapa Biosystems, Boston, MA, USA).

### Tissue preparation for immunostaining

Mice were anesthetized and transcardially perfused with saline solution containing 0.5 % sodium nitrate and heparin (10 Unit/ml), and then with 4 % paraformaldehyde in 0.1 M phosphate buffer (PB, pH 7.4). Brains were obtained and post-fixed overnight at 4 °C in 4 % paraformaldehyde. Fixed brains were stored at 4 °C in a 30 % sucrose solution until they sank. Series of coronal sections (30 μm) were obtained with a cryostat (Leica, Wetzlar, Germany), and used for immunohistochemistry.

### Immunostaining

For 3, 3′-diaminobenzidine (DAB) staining, brain sections were rinsed three times with PBS, treated with 3 % H_2_O_2_ for 5 min, and rinsed with PBS containing 0.2 % Triton X-100 (PBST). Non-specific binding was blocked with 1 % BSA in PBST. Sections were incubated overnight at room temperature with primary antibodies specific for GFAP (Neuromics, Minneapolis, MN, USA; Cat. No. RA22101). The sections were then rinsed with PBST, incubated with biotinylated secondary antibodies (Vector Laboratories, Burlingame, CA, USA), and visualized as described by the manufacturer (Vector Laboratories). Sections were mounted on gelatin-coated slides, and examined under bright field microscopy (Olympus Optical, BX51, Tokyo, Japan).

Cells were fixed with 4 % paraformaldehyde at room temperature for 20 min, washed with PBS, and incubated with 1 % BSA and 0.1 % Triton X-100 in PBS for 30 min. The cells were then incubated overnight with anti-GFAP, -TUJ-1, -MAP2, and -CNPase antibodies at 4 °C, washed with PBS, and incubated with fluorescein-conjugated secondary antibodies (Invitrogen) for 2 h. Finally, the cells were washed, mounted using a mounting medium containing 4′, 6-diamidino-2-phenylindole (DAPI; Vector Laboratories), and examined under an Axiovert 200 M microscope (Carl Zeiss, Göttingen, Germany).

### Measurement of mitochondrial-membrane potential and intracellular reactive oxygen species

NSCs were plated in 6-well plates (1.5 × 10^6^ cells/well). Mitochondrial membrane potential and intracellular reactive oxygen species (ROS) were monitored by loading cells for 30 min with 125 nM MitoTracker Red CMXRos and 10 μM carboxyl-H2DFFDA, respectively, as described previously [71]. Cells were washed twice with PBS and detached with Cellstripper TM (Media Tech, Inc., Manassas, VA, USA). Fluorescence intensities of detached cells were analyzed with a fluorescence-activated cell sorter (FACS; B-D FACS Systems, Sunnyvale, CA, USA).

### Measurement of mitochondrial DNA

For assessment of the mitochondrial DNA copy number, genomic DNA was isolated using an Exgene Cell SV kit (GeneAll, Seoul, Korea), and the content of mitochondrial DNA relative to that of nuclear DNA was measured as the ratio of the mitochondrial D-loop (mito-D-loop) to the nuclear-encoded GAPDH gene, using Q-PCR. A RotoGene thermocycler (Corbett Research, Sydney, Australia) was used with a KAP SYBR FAST qPCR kit (Kapa Biosystems, Boston, MA, USA), and the following primer pairs: mito-D-loop, 5’- CCC AAG CAT ATA AGC TAG TAC-3’ (sense) and 5’- ATA TAA GTC ATA TTT TGG GAA CTA C -3’ (antisense); and GAPDH, 5’-GCCTTCCGTGTTCCTACC-3’ (sense) and 5’-CCTCAGTGTAGCCCAAGATG-3’ (antisense). The cycle threshold (Ct) for the mito-D-loop transcript was normalized to the average Ct for GAPDH in each reaction. Relative quantification of normalized transcript abundance was performed using the comparative Ct method (ΔΔCt).

### Statistical analysis

All data presented in this study are representative of at least three independent experiments. The statistical significance of differences between mean values of two groups was assessed by the Student’s t-test. For comparisons of more than two groups, we used one-way ANOVA with Duncan’s post-hoc test.

## Additional files

Additional file 1: Figure S1. Parkin expression increased during brain development. Mouse brain lysates were collected at the indicated ages. The levels of Parkin were assayed by Western blotting. As PINK1, Parkin expression increased. (TIF 79 kb) Additional file 2: Figure S2. Confirmation of absence of PINK1 protein in PINK1 KO mice. Cell lysates were prepared from NSCs on day 5 of differentiation. PINK1 expression was analyzed with Western-blot. (TIF 61 kb) Additional file 3: Figure S3. Parkin expression increased during NSC differentiation. Cell lysates were collected on day of differentiation of NSCs. The levels of Parkin were assayed by Western blotting. As PINK1, Parkin expression increased. The data shown are representative of at least three independent experiments. (TIF 68 kb) Additional file 4: Figure S4. PINK1 deficiency did not affect the viability of NSCs during differentiation. (a) The viability of WT and PINK1-KO NSCs was examined by Western-blotting of cleaved PARP and cleaved caspase-3 on day 5 of differentiation. GAPDH was used as the loading control. The band intensities of PARP and caspase-3 were quantified (right panel). (b) LDH release was measured with an LDH-Cytotoxicity Assay Kit (Biovision, Mountain View, CA, USA). Data are presented as the means ± SEM of three samples. The data shown are representative of at least three independent experiments. (TIF 113 kb) Additional file 5: Figure S5. Blocking of protein degradation does not increase GFAP protein levels in PINK1-KO NSCs. On day 4 of differentiation, PINK1-KO NSCs were treated with the indicated amounts of a proteasomal inhibitor (MG132) or a lysosomal inhibitor (lactacystin) for 24 h, and GFAP levels were analyzed by Western blotting. The data shown are representative of at least three independent experiments. (TIF 64 kb)

## Abbreviation

GFAP: Glial fibrillary acidic protein
PD: Parkinson’s disease
PINK1: PTEN-induced kinase 1
