# Erratum to: PINK1 expression increases during brain development and stem cell differentiation, and affects the development of GFAPpositive astrocytes

**DOI:** 10.1186/s13041-016-0199-1

**Published:** 2016-02-15

**Authors:** Insup Choi, Dong-Joo Choi, Haijie Yang, Joo Hong Woo, Mi-Yoon Chang, Joo Yeon Kim, Woong Sun, Sang-Myun Park, Ilo Jou, Sang-Hun Lee, Eun-Hye Joe

**Affiliations:** Neuroscience Graduate Program Department of Biomedical Sciences, Ajou University School of Medicine, Suwon, Korea; Chronic Inflammatory Disease Research Center, Ajou University School of Medicine, Suwon, Korea; Department of Pharmacology, Ajou University School of Medicine san-5, Woncheon-dong, Youngtong-gu, Suwon, Kyunggi-do 442–721, Korea; Department of Biochemistry and Molecular Biology, College of Medicine, Hanyang University, Seoul, Korea; Department of Anatomy and Division of Brain Korea 21 Plus Biomedical Science, Korea University College of Medicine, Seoul, 136-705 Korea; Department of Brain Science, Ajou University School of Medicine, Suwon, Korea; Brain Disease Research Center, Ajou University School of Medicine, Suwon, Korea

After publication of this article [1], the authors noticed Sang-Hun Lee’s name had been misspelled as Sang Hoon Lee. The correct spelling of Sang-Hun Lee’s name is included in the author list for this erratum and has since been updated in the original article [1].
