# Supplementary figures and images for: PINK1 expression increases during brain development and stem cell differentiation, and affects the development of GFAP-positive astrocytes
Source: Mol Brain. 2016 Jan 8;9:5. doi: 10.1186/s13041-016-0186-6 (PMC4706723; doi:10.1186/s13041-016-0186-6)

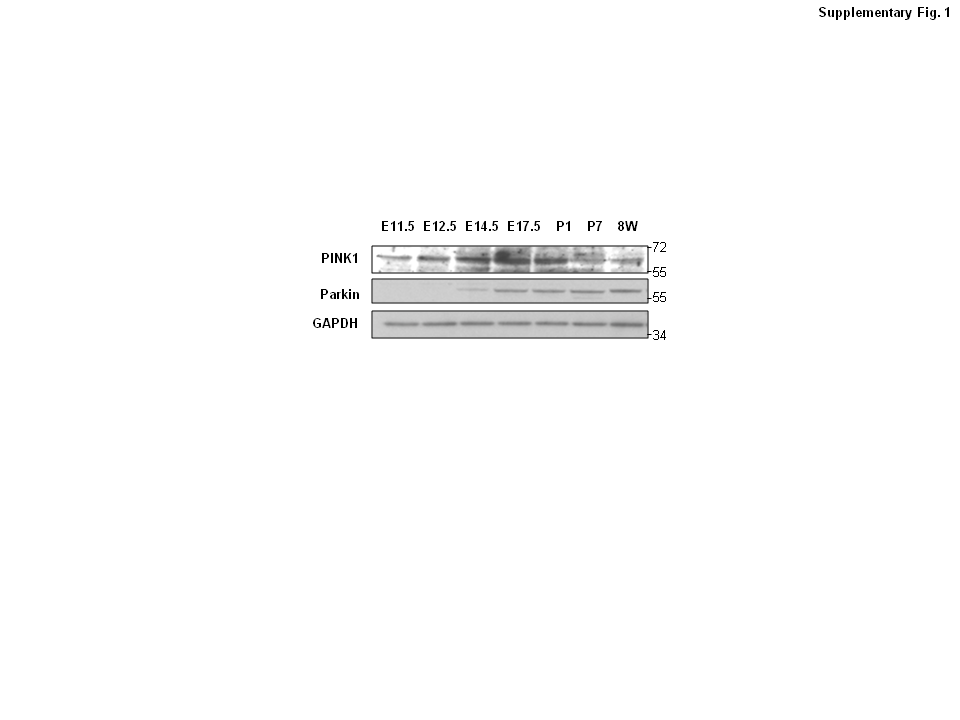

Supplement: Additional file 1: Figure S1. — Parkin expression increased during brain development. Mouse brain lysates were collected at the indicated ages. The levels of Parkin were assayed by Western blotting. As PINK1, Parkin expression increased. (TIF 79 kb) [file 13041_2016_186_MOESM1_ESM.tif]

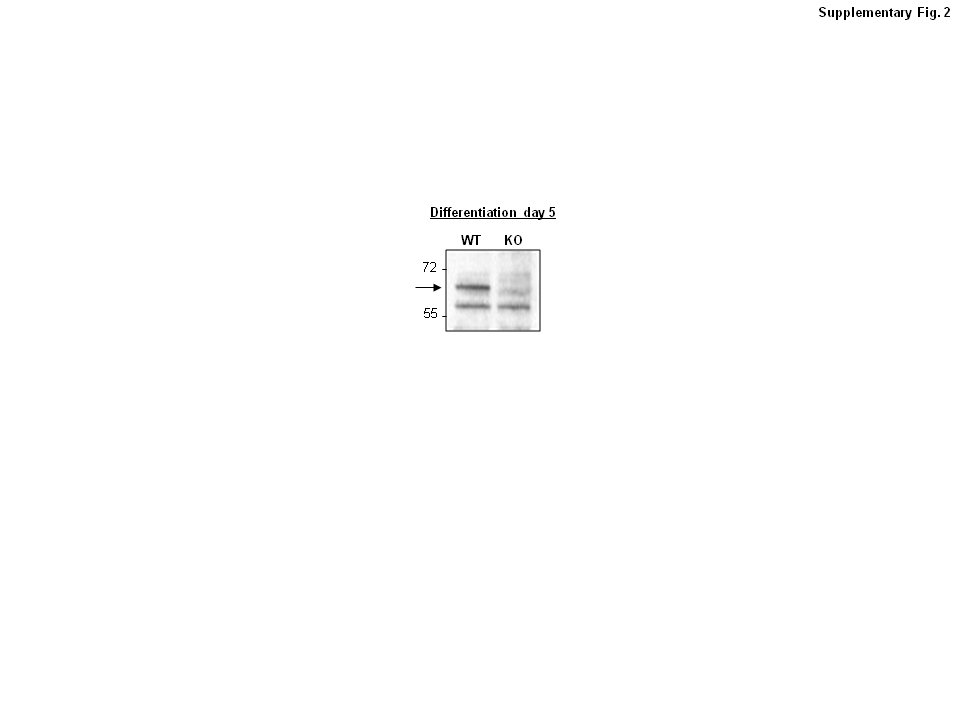

Supplement: Additional file 2: Figure S2. — Confirmation of absence of PINK1 protein in PINK1 KO mice. Cell lysates were prepared from NSCs on day 5 of differentiation. PINK1 expression was analyzed with Western-blot. (TIF 61 kb) [file 13041_2016_186_MOESM2_ESM.tif]

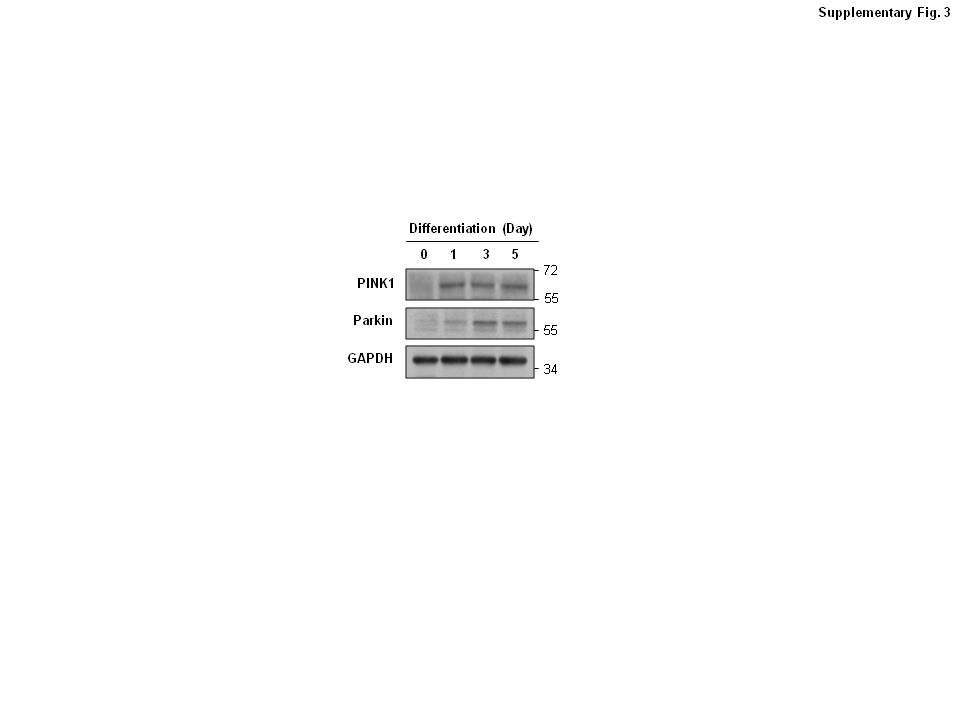

Supplement: Additional file 3: Figure S3. — Parkin expression increased during NSC differentiation. Cell lysates were collected on day of differentiation of NSCs. The levels of Parkin were assayed by Western blotting. As PINK1, Parkin expression increased. The data shown are representative of at least three independent experiments. (TIF 68 kb) [file 13041_2016_186_MOESM3_ESM.tif]

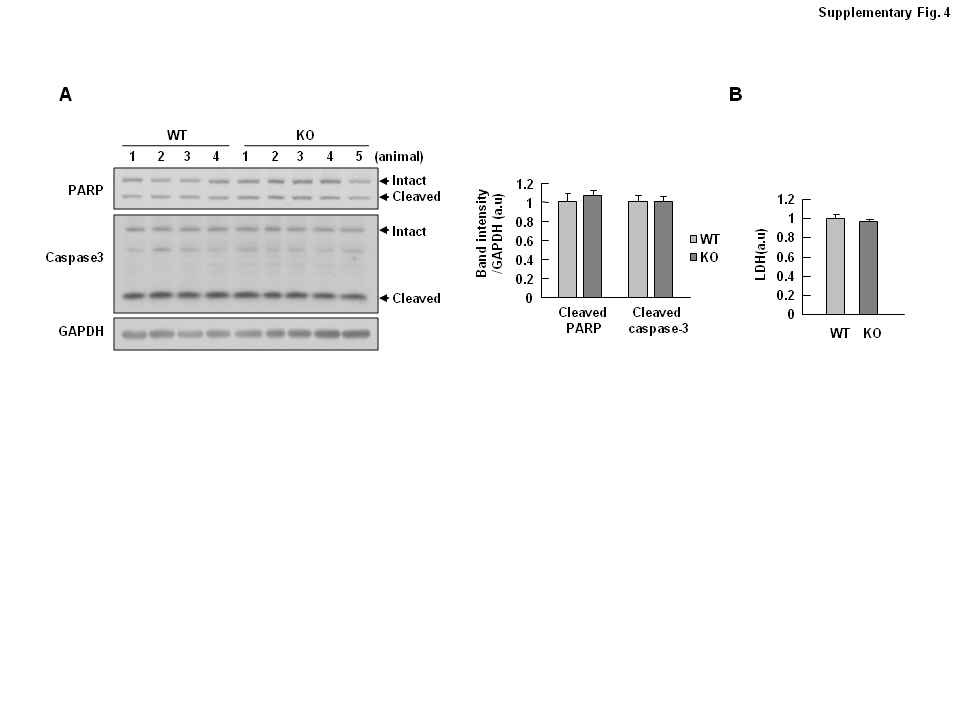

Supplement: Additional file 4: Figure S4. — PINK1 deficiency did not affect the viability of NSCs during differentiation. (a) The viability of WT and PINK1-KO NSCs was examined by Western-blotting of cleaved PARP and cleaved caspase-3 on day 5 of differentiation. GAPDH was used as the loading control. The band intensities of PARP and caspase-3 were quantified (right panel). (b) LDH release was measured with an LDH-Cytotoxicity Assay Kit (Biovision, Mountain View, CA, USA). Data are presented as the means ± SEM of three samples. The data shown are representative of at least three independent experiments. (TIF 113 kb) [file 13041_2016_186_MOESM4_ESM.tif]

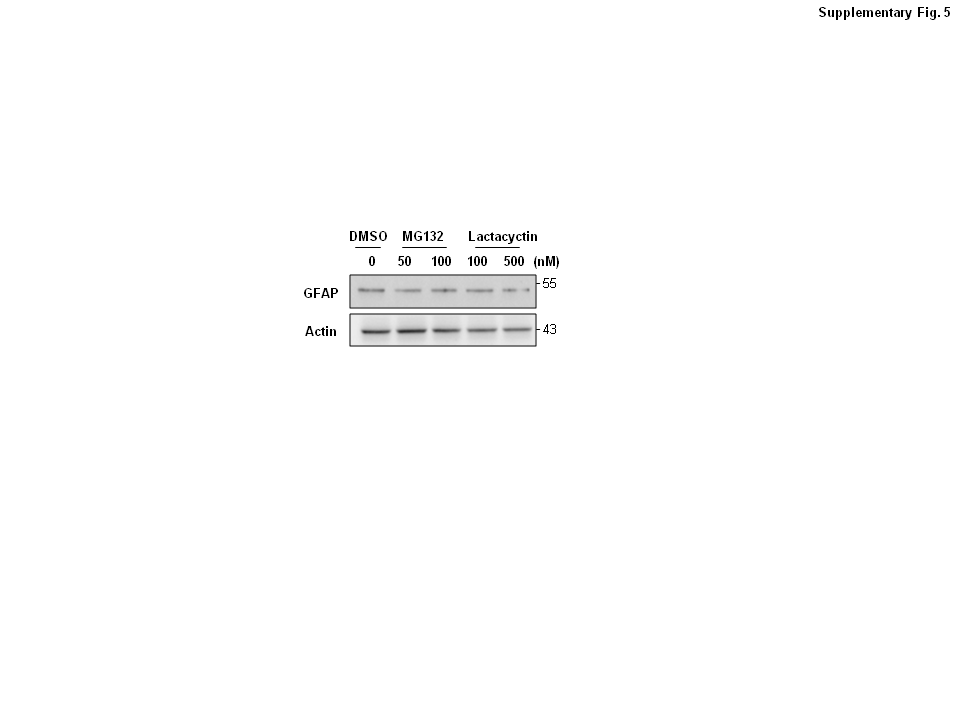

Supplement: Additional file 5: Figure S5. — Blocking of protein degradation does not increase GFAP protein levels in PINK1-KO NSCs. On day 4 of differentiation, PINK1-KO NSCs were treated with the indicated amounts of a proteasomal inhibitor (MG132) or a lysosomal inhibitor (lactacystin) for 24 h, and GFAP levels were analyzed by Western blotting. The data shown are representative of at least three independent experiments. (TIF 64 kb) [file 13041_2016_186_MOESM5_ESM.tif]
